# The creation of the Belmont Report and its effect on ethical principles: a historical study

**DOI:** 10.1007/s40592-022-00165-5

**Published:** 2022-11-10

**Authors:** Hiroyuki Nagai, Eisuke Nakazawa, Akira Akabayashi

**Affiliations:** 1 Department of Biomedical Ethics, University of Tokyo Faculty of Medicine, 7-3-1 Hongo, Bunkyo-ku, Tokyo, 113-0033 Japan; 2 Center for Technology Research and Innovation, BIPROGY Inc, 1-1-1 Toyosu, Koto-ku, Tokyo, 135-8650 Japan; 3 Division of Medical Ethics, New York University School of Medicine, 227 East 30th Street, New York, NY 10016 USA

**Keywords:** Belmont Report, Beneficence, Common Rule, Ethical principles, Gene therapy, Justice, Respect for Persons, United States

## Abstract

The Belmont Report continues to be held in high regard, and most bioethical analyses conducted in recent years have presumed that it affects United States federal regulations. However, the assessments of the report’s creators are sharply divided. Understanding the historic reputation of this monumental report is thus crucial. We first recount the historical context surrounding the creation of this report. Subsequently, we review the process involved in developing ethical guidelines and describe the report’s features. Additionally, we analyze the effect of unfolding events on the subsequent creation of federal regulations, especially on gene therapy clinical trials. Moreover, throughout this paper we evaluate the ethical principles outlined in this report and describe how they overlap with the issue of protecting socially vulnerable groups. Based on the analysis, we conclude that the features of the Belmont Report cannot be considered as having affected the basic sections of the federal regulations for ethical reviews that were made uniform in 1981. Nevertheless, regarding the regulations on gene therapy clinical trials—which were at first expected to be applicable to research involving children—in addition to implementing policies regarding the public review of protocols that passed ethical review, this report’s principles are clearly reflected in the key notes that should have been referred to when the report was created.

## Introduction

‘The Belmont Report: Ethical Principles and Guidelines for the Protection of Human Subjects of Research’ (National Commission 1979), which was written by the National Commission for the Protection of Human Subjects of Biomedical and Behavioral Research (hereinafter, National Commission) and publicly listed in the Federal Register in April 1979, is one of the basic documents related to bioethics policy in the United States. This report was written after the Department of Health, Education, and Welfare (DHEW; now the Department of Health and Human Services [HHS]), in accordance with the National Research Act (enacted in July 1974), created the National Commission to examine and identify comprehensive ethical principles for the purpose of protecting human subjects. One of the results of this effort was a request that the report reflect the federal regulation known as ‘Protection of Human Subjects (45 CFR 46)’ (DHEW 1974), which the DHEW had already publicly released in May 1974. The core of the report was principles—Respect for Persons, Beneficence, and Justice—and their specific applications (Informed Consent, Assessment of Risks and Benefits, and Selection of Subjects).

Nevertheless, despite research into the Belmont Report progressing in the twenty first century (Bertholf 2001; Breault 2006; Friesen et al. 2017; Porter and Koski 2008), opinions remain divided over the effect the report has actually had on policies for the protection of human subjects in the United States. At the time of its creation, the members of the National Commission, the staff philosophers, and the staff directors harbored differing views on the influence the Belmont Report would have. The 20th and 25th anniversaries of the release of the report provided opportunities to reflect on its historical significance (Childress et al. 2005; Fox and Swazey 2008). In particular, the 2004 Oral History of the Belmont Report and the National Commission for the Protection of Human Subjects of Biomedical and Behavioral Research (hereinafter, Oral History) reflected a trend of contrasting views on its effect (Oral History 2004). Theologians and commissioners Albert R. Jonsen and LeRoy Walters, physician and commissioner Duane Alexander, staff philosopher Stephen Toulmin, and National Institute of Health (NIH) liaison Charles R. McCarthy recognized the effect of this report on federal regulations. Walters pointed out that this reflected the regulations for gene therapy clinical trials through deliberation related to the President’s Commission for the Study of Ethical Problems in Medicine, Biomedical, and Behavioral Research (hereinafter, President’s Commission). Alexander also mentioned the possibility that its publication may have a certain degree of influence on regulations that would be subsequently announced. In contrast, physician and commissioner Robert Levine, staff philosopher Tom L. Beauchamp, lawyer and staff director Michael Yesley, and assistant director Barbara Mishkin did not fully recognize the effect the report would have on federal regulations but indicated that the report was intended to provide only a general moral framework. Levine’s assessment was that the report would not lay the foundation for regulations at the Food and Drug Administration (FDA), which has jurisdiction over clinical trials with human subjects conducted by the DHEW and affiliated organizations. Yesley and Mishkin, as far as we understand, opined that the Belmont Report would not be related to individual regulations, as it was merely an expression of general criteria.

One of the most important features of the Belmont Report is its three ethical principles. Regarding them, in addition to further refining our assessment of this historical document, it is important to look back on the influence the creation of these principles had on the subsequent establishment of related policies. The history of the development of bioethics can be considered as a process of developing criticism among multiple theories regarding ethical principles. One of the basic issues taken up was whether the relationship among the multiple principles should be focused on Autonomy or Beneficence (Veatch 1995).

Beauchamp first published *Principles of Biomedical Ethics* in 1979 with James F. Childress. Four principles were proposed in this book: Autonomy, Beneficence, Non-maleficence, and Justice (Beauchamp and Childress 1979). H. Tristram Engelhardt developed a theory that prioritized principles related to Autonomy or Liberty over Beneficence (Engelhardt 1996). However, Edmund D. Pellegrino placed importance on Beneficence as the only principle connected to medical practices (Pellegrino and Thomasma 1988). Hence, there arose views criticizing a principles-based approach to the creation of theories combining multiple obligations that lack absoluteness (DeGrazia 1992). Bernard Gert and K. Danner Clouser advocated the theory of Common Morality and emphasized the principle of Beneficence as a moral rule opposed to the abuse of Freedom (Clouser and Gert 1990; Gert et al. 2006). While these two ideas were organized as representative arguments (Richardson 1990, 2000), Beauchamp and Childress subsequently adopted a position in which they maintained conventional views while simultaneously accepting the theory of Common Morality (Beauchamp 1995, 2007; Beauchamp and Childress 2019).

It is possible that, by placing it within the flow of these kinds of arguments, the Belmont Report could have been applied increasingly more broadly without having paid sufficient attention to the historical and cultural background that led to its creation (Beauchamp 2006; Cassell 2000; Friesen et al. 2017; Miller 2006). While this report should be widely applied to all research involving human subjects, there is some criticism that the report doesn’t sufficiently account for the different circumstances in behavioral science research. Indeed, the legacy of the National Commission should be approached in a way that allows historians to contribute to the academic discussions by philosophers and physicians (Brothers et al. 2019).

Hence, in this paper, bioethics experts in history, philosophy, and medicine jointly address this issue. First, we summarize the historical context up to the point at which there arose a need to create the report. Second, we reflect on the process by which the National Commission created the ethical principles and describe their features. Finally, we analyze the influence these characteristics had on the creation of related regulations by the DHEW and the FDA following the publication of the report as well as the gene therapy clinical trials conducted by the NIH.

## Pre-Belmont: historical context of ethical principles for the protection of human subjects

The medical ethics code often referred to as the Nuremberg Code was decreed in August 1947 by the judges in the International Military Tribunal, Case One, widely known as the Doctors’ Trial. The Code specified a requirement of “voluntary consent” as an essential requirement of human experiments. The voluntary consent requirement can be seen as an application of the principle of Respect for Autonomy, although the principle itself wasn’t explicitly mentioned in the trial (Schmidt 2004). The ‘Declaration of Helsinki: Recommendations Guiding Doctors in Clinical Research’, adopted in its first version in June 1964 by the World Medical Association (WMA), emphasized the ethical principle of Beneficence. However, neither of these documents fully considered the participation in research of socially vulnerable groups such as children or adults who don’t have the capacity to make decisions. In particular, the former had the limitation that it was drafted in reference to prisoners in the unique circumstances at the concentration camps. Consequently, the US Congress instructed the DHEW to identify comprehensive ethical guidelines that included such groups and the DHEW acted upon these instructions.

The Nuremberg Code positions the voluntary consent of subjects as the “essential” condition for participation in clinical research (The Nuremberg Code 1949). The first of the Code’s ten ethical principles is the absolute necessity of voluntary consent by human subjects who are legally competent to make decisions and are free to do so without coercion. Although the Code includes elements that correspond to the principles of Beneficence and Justice, it is focused on Autonomy itself (Grodin 1992). In contrast to the Nuremberg Code, the Declaration of Helsinki entrusted to a research ethics committee (in the U.S., an institutional review board or IRB) the decision on whether a research study is approvable apart from the importance placed on the autonomy of human subjects, which is emphasized in the Nuremberg Code (Fluss 1999; Roman 2002).

In October 1962, the WMA publicized a draft of its declaration in which it distinguished medical research into (1) Experiments for the Benefit of the Patient and (2) Experiments Conducted Solely for the Acquisition of Knowledge (Draft Code of Ethics on Human Experimentation 1962). In the first, participation in research is possible as long as the subject him- or herself, a relative, or a legal guardian grants consent, while the second points out the difficulty in protecting socially vulnerable groups such as children and adults who don’t have the capacity to make decisions for themselves. Hugh Clegg, an editor at the *British Medical Journal* who was involved in the framing of this draft declaration, based the adoption of proxy consent not upon the Nuremberg Code, but rather on Hippocratic medicine, namely on a principle of Beneficence (Annas 1991). In the final version of 1964, this provision was changed to the following: (1) Clinical Research Combined with Professional Care and (2) Non-therapeutic Clinical Research (Human Experimentation 1964). Thus, the framework for the protection of socially vulnerable groups was left vague. The Declaration was revised in October 1975 to require that not only clinical research but also protocols for non-clinical biomedical research be transmitted for ethical review ( WMA  1976).

The DHEW began preparing guidance and related federal regulations in order to allow socially vulnerable groups to participate in research following the adoption of the Declaration of Helsinki. Under the auspices of the DHEW, the Public Health Service (PHS)—which had jurisdiction over the NIH—released a policy statement in July 1966. In this statement, the PHS established a policy of not requiring the consent of the subject him- or herself in cases in which the participation of legally incompetent persons in the experimental procedure may be of benefit to themselves (Gray 1975). In the wake of this position, the NIH released a guide to be used by institutions to implement the policy (NIH 1971). This manual indicated that one issue was the fact that careful consideration must be paid to how socially vulnerable groups are handled, while at the same time, it adopted the principles of both the Nuremberg Code and the Declaration of Helsinki. In October 1973, the DHEW proposed the 45 CFR 46 basic policy and announced that subparts on the protection of these groups were to be additionally established (DHEW 1973). In May 1974, the final version of this basic policy was publicly disclosed in the Federal Register (DHEW 1974).

The US Congress established the National Research Act in July 1974. The National Commission was set up in the DHEW, and it conducted a survey of the comprehensive ethical principles related to biomedical and behavioral research. Based on the results of this survey, a request was issued to establish a federal policy for protecting human subjects. The DHEW planned to announce the following in order: The 45 CFR 46 basic policy, in subpart A; those specifically for fetuses, pregnant women, and in vitro fertilization, in subpart B; those for prisoners, in subpart C; those for children, in subpart D; and those for those institutionalized as mentally infirm, in subpart E. In August of that year, a proposal was made for subpart B, and the National Commission had already indicated the following three principles as transitional measures until the ethical principles demanded by the US Congress were identified:

(1) To avoid harm whenever possible, or at least to minimize harm; (2) to provide for fair treatment by avoiding discrimination between classes or among members of the same class; and (3) to respect the integrity of human subjects by requiring informed consent. (DHEW 1975, p. 33545)

These measures highlight how the idea of Non-maleficence and the principle of Justice can be distinguished from the principle of Beneficence, and these were not fully elucidated in the principles of the Nuremberg Code and the Declaration of Helsinki. This subpart was publicly released in August 1975 (Table 1).

**Table 1:** Chronological table of the formation of the principles of the Belmont Report of the Department of Health, Education, and Welfare (1973–1983)

Year/month	Office of the secretary	National Commission for the protection of human subjects of biomedical and behavioral research
1973/10	Protection of Human Subjects (45 CFR 46) subpart A (Basic Policy): Proposed	
1974/5	Protection of Human Subjects (45 CFR 46) subpart A (Basic Policy): Publicly released	
1974/7		National Commission: Established
1974/8	Protection of Human Subjects (45 CFR 46) subpart B (Fetuses, Pregnant Women, and In Vitro Fertilization): Proposed	
1975/8	Protection of Human Subjects (45 CFR 46) subpart B (Fetuses, Pregnant Women, and In Vitro Fertilization): Publicly released	
1976/2		Belmont Conference: Commenced
1977/9		Report on Research Involving Children: Submitted to the President
1978/6		Draft of the Belmont Report: Ethical Principles and Guidelines for the Protection of Human Subjects of Research: Approved
1978/7	Protection of Human Subjects (45 CFR 46) subpart D (Children): Proposed	
1978/9		Report on Institutional Review Boards: Submitted to the President
1978/9		Belmont Report: Submitted to the President
1979/4		Belmont Report: Publicly released
1979/8	Regulations Amending Basic HEW Policy for Protection of Human Research Subjects (45 CFR 46) subpart A: Re-Proposed	
1981/1	Regulations Amending Basic HEW Policy for Protection of Human Research Subjects (45 CFR 46) subpart A: Revised	
1983/3	Protection of Human Subjects (45 CFR 46) subpart D (Children): Publicly released	

## The creation of the Belmont Report

From this point onwards, we will reflect on the process of creating the Belmont Report and summarize the features of its ethical principles. Up to this point, the fact that balance between the principles was lacking had been criticized, but the three principles were created in such a way that their implications would overlap with the protection of socially vulnerable groups in mind.

At the Belmont Conference held in February 1976, the National Commission established three ethical principles, and based on these, it created secondary principles that would overlap around the issue of the protection of socially vulnerable groups (National Commission 1976). At this Conference, theologian and commissioner Karen Lebacqz proposed seven principles. These were: (1) Respect for the self-determination of human beings, (2) Concern to benefit individual human research subjects, (3) Concern to benefit other individuals and groups (including both present and future benefits), (4) Concern to minimize harm to individual research subjects, (5) Concern to minimize the consequential harm to others, (6) Concern for distributive justice, and (7) Concern for compensatory justice. Following this, Toulmin, the staff philosopher, proposed adding (8) The protection of the weak, the powerless. Commissioner Joseph Brady, a psychiatrist, simplified these protections into three—(1) Beneficence, which would be most benefit and least harm, (2) Freedom, minimize coercion and maximize self-determination, and (3) Justice, equal protection and equal opportunity; and equity—and requested that eight principles be organized into secondary principles (National Commission 1976, pp. 109–110). Toulmin drew up the first draft of the report after the conclusion of the Belmont Conference, and Beauchamp, his successor as the staff philosopher, made partial revisions to the sections related to Justice by December 1977 (Beauchamp 2020; Nukaga 2019). They encouraged the commissioners to specify that Respect for Persons was fundamentally Respect for Autonomy, but many commissioners interpreted the ‘Freedom’ included in Brady’s proposal as ‘Respect for Persons’ as they continued their discussions of the three principles of Beneficence and Justice (Oral History 2004: Interview with Albert R. Jonsen), and did not accept this idea. Levine and Walters argued that in Western philosophy, Autonomy was an idea that belonged to the most recent tradition (Oral History 2004: Interview with Walters and Robert Levine).

Jonsen, Beauchamp, and Toulmin received approval for their wording from the National Commission in June 1978 (Jonsen 1998). This version included the two implications that Respect for Persons was a matter of ‘Autonomy’ and ‘The protection of the weak, the powerless’ and that Beneficence was ‘Concern to benefit’ and ‘Concern to minimize harm’, and it established Distributive Justice (selection between the beneficiary and the burdened) as the principle linking the two. Beauchamp said that he believed ethical principles were not a tool to achieve balance, but rather, that they were directly linked, and for that reason, the accommodation mentioned above was insufficient (Oral History 2004: Interview with Tom L. Beauchamp). In particular, Respect for Persons is included in at least two ethical convictions. The first is the idea that individuals should be treated as autonomous actors and the second is the idea that those who have lost autonomy have the right to be protected. However, the idea of respect for personhood of children or legally incompetent adults is in in practice similar to the requirements of the Beneficence principle, and therefore, there was concern that there may be dependence upon risks that cause harm (National Commission 1978a). Despite this, however, commission chair Kenneth Ryan as well as commissioners Dorothy Height, Patricia King, David W. Louisell, and Donald Seldin advocated the need to attempt to appropriately balance these principles based on the idea that ethical reviews of research involving children may put these principles into conflict with one another (National Commission 1977, pp. 123–133). The National Commission submitted the final version of the Belmont Report to the President in September of that year (National Commission 1978c).

## Post-Belmont: effects on immediate US federal regulations

Following publication in April 1979, the contents of the Belmont Report in themselves did not have an effect on the fundamentals of the DHEW’s 45 CFR 46 regulations on the protection of human subjects. Through the process of formulating ethical review regulations in a comprehensive framework that would accommodate the demands of both the DHEW, which managed research grants, and the FDA, which demanded compliance with federal laws, the initial requirement included positioning the principles that contained the Nuremberg Code and Declaration of Helsinki, and the rules unique to each relevant institution. However, this report was not named. Nevertheless, it was mentioned subsequently during the process of forming the ‘Basic HHS Policy for Protection of Human Research Subjects’ in 1981 (HHS 1981).

By 1981, the FDA standardized the scope of research targeted for review to align it with the scope used by the DHEW. Although the FDA had published its policy on clinical trials involving human subjects in July 1966 (FDA 1966), the WMA, US Congress, and DHEW demanded that the ethical review regulations be coordinated. Hence, in August 1978, the ‘Standards for Institutional Review Boards for Clinical Investigations’ (21 CFR 56), which were ethical review regulations that referred to 45 CFR 46, were proposed (FDA 1978). However, in its ‘Institutional Review Boards’ report submitted to the US President in September, the National Commission criticized this in terms of protecting the best interests of human subjects. (National Commission 1978b, pp. 103–104). In November that same year, the FDA was informed that this report was published in Federal Register, and it withdrew its proposal. In August 1979, 21 CFR 56 was re-proposed that was consistent with that of the DHEW (FDA 1979a, b). In addition to acknowledging that there was a gap with the DHEW, which reviewed biomedical and behavioral research prior to providing them with grant money, as IRBs review clinical trial protocols in the field of biomedicine based on federal regulations, IRBs presented a policy of aiming for a comprehensive framework that would allow prior review of clinical trial protocols. The DHEW also proposed revising subpart A of 45 CFR 46 at the same time as the FDA re-proposal. The expected outlook was that regulations would be relaxed in order to facilitate exempting from review research proposals that were thought to involve nearly no risk to human subjects (e.g., social, economic, and educational surveys) and the inclusion of biomedical research as the main targets of ethical review (DHEW 1979). The final versions of the DHEW and FDA regulations were both publicized in January 1981.

In the 1971 NIH’s institutional guide to DHEW policy, the DHEW required that the points IRBs needed to comply with took into account existing codes and declarations. However, initially, this was not thought to require any connection to prospects related to assessments of the risk to human subjects (NIH 1971). This policy underwent major changes during the process of creating the ‘Federal Policy for the Protection of Human Subjects’ (hereinafter, Common Rule) in the 1980s. In order to make exemptions for research that entailed nearly no risk, it would probably be necessary to conduct a thorough investigation of their relation to research that entailed risks. In its biennial report issued in December 1981, the President’s Commission, established in November 1978, recommended that the creation of federal regulations be undertaken as the common core of 45 CFR 46 subpart A in January 1981 (President’s Commission 1981). The Office of Science and Technology Policy (OSTP) of the Executive Branch proposed a common basic policy in June 1986, and in November 1988, a common regulation proposal was released by an interagency committee. When the Common Rule was drawn up in June 1991 (Department of Agriculture et al. 1991), it was recognized that special attention was required for assessments of risk that would be experienced by socially vulnerable groups, under the Belmont Report’s ethical principles of protecting human subjects.

## Post-Belmont: effects on regulations of gene therapy clinical trials

The ethical principles of the Belmont Report were reflected in the de facto considerations for the regulations for gene therapy clinical trials that were hoped to be applied as soon as possible to children. The National Commission proposed conducting public reviews that considered the three principles in the report on research involving children, the President’s Commission requested that 45 CFR 46 subpart D be submitted to HHS as soon as possible, and the NIH followed the process up to the point at which the considerations for conducting relevant clinical trials that reflected these were created.

The application of ethical principles was assumed a part of the review of research involving children. In the report known as ‘Research Involving Children’, which was submitted to the US President in September 1977, the National Commission included the Belmont Report’s three ethical principles of Respect for Persons, Beneficence, and Justice (Jonsen 1978). Recommendation 6 required that a national bioethics commission conduct a public review to determine whether a research protocol violated the three principles in cases that an IRB determined that it was impossible to review appropriately. The report included a statement by several commissioners, including chair Ryan, explaining three ethical principles (National Commission 1977). Mention is made of the facts that the autonomous decisions of children are not clarified in the principle of Respect for Persons, that Beneficence requires a rational framework for the utilization of children, and that Justice requires that attention be paid to whether children*—*who are easy to utilize in research*—*are exhausted by their participation. The division of the roles of IRBs and national bioethics commissions is indicated. In July 1978, the DHEW proposed subpart D of 45 CFR 46 for children, in which a comprehensive review mechanism was adopted that invokes public review at the national level under exceptional circumstances, while also emphasizing that IRB reviews would play the main role (DHEW 1978). National bioethics commissions would not be permanent bodies. Rather, it would be desirable to set up a panel of experts that would support national research projects. Recommendation 5 of the biennial report published by the President’s Commission in December 1981 contained a requirement that HHS take the step publicized in the final version of 45 CFR 46 subpart D as soon as possible (President’s Commission 1981). In a public hearing held in September 1982, the Commission requested swift implementation by HHS Director Richard Schweiker (President’s Commission 1983). HHS publicly announced the final version in March 1983 (HHS 1983).

Examples of regulations on gene therapy clinical trials were presented as the kind of ‘exceptional circumstances’ that would apply, and seven points that would be adapted to the three principles of the Belmont Report were indicated (Walters and Palmer 1997). The President’s Commission understood the risks involved in a draft of the report on such trials that included a situation in which the judgment of the parents was to be secured (Powledge 1980), and it had a negative stance toward proxy consent based on the view that it would increase the parents’ responsibility as gene therapies advanced. In November 1982, the Commission published a report entitled ‘Splicing Life’, in which it recommended that the Recombinant DNA Advisory Committee (RAC) of the NIH, which established the ‘Guidelines for Research Involving Recombinant DNA Molecules’ in June 1976 (Nagai et al. 2009), play a role in the expert panel that would conduct public reviews (President’s Commission 1982). The RAC held a meeting in September 1983 and determined a policy according to which bioethics experts would be included in the membership and would take on the responsibility of public reviews required by 45 CFR 46 (NIH 1984). This expert panel was known as the ‘Working Group on Human Gene Therapy’. Its members included former committee member of the National Commission as well as Walters, and they began de facto deliberations on the important considerations with which they were charged (Areen 1985).

In January 1984, the NIH proposed revisions to the existing recombinant DNA guidelines. and released its ‘Points to Consider in the Gene Therapy Protocols’ (Working Group on Human Gene Therapy 1985). The points to consider required that gene therapy clinical trials be conducted in accordance with 45 CFR 46 subparts A and D, and it indicated the following seven points to be adapted with the three principles: (1) What is the disease to be treated? (2) What alternative interventions are available for the treatment of this disease? (3) What is the anticipated or potential harm of the experimental gene therapy procedure? (4) What is the anticipated or potential benefit of the experimental gene therapy procedure? (5) What procedure will be followed to ensure fairness in the selection of patient-subjects? (6) What steps will be taken to ensure that patients, or their parents or guardians, give informed and voluntary consent to participating in the research? Finally, (7) How will the privacy of patients and the confidentiality of their medical information be protected? (Walters and Palmer 1997, pp. 37–44) In July 1990, the NIH approved a clinical trial of patients with adenosine deaminase (ADA) deficiency as its first case and implementation of the trial was entrusted to the institution.

## Conclusion

In this paper, we attempted a historical evaluation of the effect the Belmont Report had on federal regulations regarding the protection of human subjects of research. The ethical principles mentioned in the Belmont Report linked the various protections of those in socially vulnerable groups and included secondary principles. Its characteristics did not have an effect on the basic parts of the common ethical review regulations established by the DHEW and FDA in January 1981. Nevertheless, regarding the regulations for gene therapy clinical trials—which was hoped to be applied as soon as possible to children—a policy was created that included public review by the NIH of protocols following an ethical review, and the points to consider that needed to be referred to when creating a research protocol were reflected clearly in the report’s principles.
